# Hypoxically stored RBC resuscitation in a rat model of traumatic brain injury and severe hemorrhagic shock^☆^

**DOI:** 10.1016/j.lfs.2024.122423

**Published:** 2024-01-25

**Authors:** Cynthia R. Muller, Vasiliki Courelli, Krianthan Govender, Laurel Omert, Tatsuro Yoshida, Pedro Cabrales

**Affiliations:** a Functional Cardiovascular Engineering Laboratory, Bioengineering Department, UC San Diego, La Jolla, CA 92093, United States of America; b Hemanext, Lexington, MA, United States of America

**Keywords:** TBI, Hemorrhagic shock, Hypoxic RBCs

## Abstract

This study aims to investigate the effects of hypoxically stored Red Blood Cells (RBCs) in a rat model of traumatic brain injury followed by severe hemorrhagic shock (HS) and resuscitation.

RBCs were made hypoxic using an O_2_ depletion system (Hemanext Inc. Lexington, MA) and stored for 3 weeks. Experimental animals underwent craniotomy and blunt brain injury followed by severe HS. Rats were resuscitated with either fresh RBCs (FRBCs), 3-week-old hypoxically stored RBCs (HRBCs), or 3-week-old conventionally stored RBCs (CRBCs). Resuscitation was provided via RBCs transfusion equivalent to 70 % of the shed blood and animals were followed for 2 h. The control group was comprised of healthy animals that were not instrumented or injured.

Post-resuscitation hemodynamics and lactate levels were improved with FRBCs and HRBCs, and markers of organ injury in the liver (Aspartate aminotransferase [AST]), lung (chemokine ligand 1 [CXCL-1] and Leukocytes count), and heart (cardiac troponin, Interleukin- 6 [IL-6] and Tumor Necrosis Factor Alpha[TNF-α]) were lower with FRBCs and HRBCs resuscitation compared to CRBCs. Following reperfusion, biomarkers for oxidative stress, lipid peroxidation, and RNA/DNA injury were assessed. Superoxide dismutase [SOD] levels in the HRBCs group were similar to the FRBCs group and levels in both groups were significantly higher than CRBCs. Catalase levels were not different than control values in the FRBCs and HRBCs groups but significantly lower with CRBCs. Thiobarbituric acid reactive substances [Tbars] levels were higher for both CRBCs and HRBCs. Hypoxically stored RBCs show few differences from fresh RBCs in resuscitation from TBI + HS and decreased organ injury and oxidative stress compared to conventionally stored RBCs.

## 1. Introduction

Trauma and hemorrhage remain one of the leading causes of mortality in the USA, representing a significant problem to the health care system [1]. Despite the many benefits of expeditious transfusion in hemorrhaging trauma patients, observational studies have reported adverse outcomes associated with transfusion of red blood cells (RBCs) [2–4]. Some of these adverse outcomes may be a result of structural or biochemical changes that RBCs undergo while in storage, which are frequently referred to as the “storage lesions” [5,6]. Storage lesions are described as metabolic, oxidative, chemical, and structural changes occurring in RBCs during processing, storage, and preparation for transfusion [7–9]. Prior research has shown that damage to RBCs caused by storage becomes significant after two weeks [10,11], despite the current guidelines stipulating that RBCs can be stored for 35 and 42 days depending on the additive used for storage [12]. Multiple mechanisms of the RBCs storage lesions have been described that ultimately lead to systemic inflammation, end-organ damage, and coagulopathy [13]. Among several hypotheses, the oxidative stress/free-radical theory offers the best mechanistic explanation of in-vitro aging of RBCs [14]. These changes have been associated with increased storage time and data suggests that transfusion of aged RBCs as opposed to fresh RBCs may influence clinical outcomes [6,13,15,16].

A previous study conducted by our group in a rat model of severe hemorrhagic shock (HS) demonstrated that resuscitation with hypoxically stored RBCs resulted in recovered cardiac function, restored hemodynamic stability, reduced lactate levels, and improved overall outcomes. Notably, effective resuscitation using hypoxically stored blood was achieved in half the time and required only half the volume of conventionally stored RBCs [17]. The present study aims to evaluate the effectiveness of hypoxically stored RBCs in the resuscitation of traumatic brain injury (TBI) combined with severe HS. We hypothesize that hypoxic storage of RBCs can enhance their quality and, consequently, provide comparable or superior efficacy compared to fresh blood when used for resuscitation in severe TBI + HS scenarios.

## 2. Methods

### 2.1. Blood donation

Male Sprague Dawley donor rats (400–450 g) (Charles River Laboratories Wilmington MA), were anesthetized using isoflurane 5 % in compressed room air for induction (Drägerwerk AG, Lübeck, Germany) and then maintained at 2.5 %. Animals were placed on a heating pad to preserve core body temperature at 37 °C and allowed to freely breathe from a nosecone delivering anesthesia. Blood was drained using a heparinized 18G needle and syringe via cardiac puncture under isoflurane anesthesia. Once blood was drained, the animal was euthanized with Euthasol (sodium pentobarbital, 300mg/kg).

### 2.2. Preparing blood for storage

The blood from the donor animals was pooled into 50 mL conical tubes containing 0.14 mL of Citrate Phosphate Double Dextrose (CP2D, Haemonetics Corp, Union, SC) for every 1 mL of whole blood. The tubes were then soft spun at ~1000 G (2600 RPM on an IEC Centra CL2 Centrifuge) to allow for removal of the topmost layer containing platelet rich plasma (PRP) in each of the tubes. Blood was then pooled in a 150 mL Fenwal 4R2001 transfer bag (Fresenius Kabi, Bad Homburg, Germany) containing 0.22 mL of Additive Solution-3 (AS-3, Haemonetics Corp, Union, SC) for every 1 mL of whole blood. The Fenwal transfer bag was connected to a NEO High-Efficiency Leukocyte Reduction Filter (Haemonetics Corp, Union, SC) for leukodepletion. For the conventionally stored red blood cells (CRBCs), the resultant RBCs were then stored in a modified PVC transfer bag (Fenwal Cat# 4R2001) sealed to reduce capacity to ~50 mL) until samples were needed. For the hypoxic/hypocapnic stored red blood cells (HRBCs), extra processing steps reduced pO_2_ and thereby hemoglobin oxygen saturation (SO_2_), as well as pCO_2_. After leukodepletion, the RBCs were then transferred to a modified Hemanext One^®^ Oxygen Reduction Bag (ORB) and agitated by a linear table shaker driven by a NSH-34RH motor (Bodine Electric Company, Northfield, IL) controlled by a MotorMaster 20,000 series adjustable speed drive (Minarik Electric Company, Glendale, CA) set to 60 RPM for 5 h at room temperature. Samples were taken every 15 min from the bag to measure changes in SO_2_ and blood gases over the course of deoxygenation. The deoxygenated RBCs were then transferred to modified PVC transfer bags, which were sealed inside oxygen barrier bags (PackFreshUSA; quart size) along with oxygen sorbent packets (AGELESS^®^ Mitsubishi Gas Chemical América (MGC) GLS; capacity of 500 mL) to scavenge residual oxygen and carbon dioxide from the bag during storage. Both bags containing CRBCs and HRBCs were stored for 3 weeks in a refrigerator set to 4 °C. Fresh red blood cells (FRBCs) were processed in the same manner as the CRBCs but were transfused within 72 h after processing. Prior to transfusion, RBCs were analyzed with ABL90 FLEX blood analyzer (Radiometer, Copenhagen, Denmark) for SO_2_, blood gases, pH, glucose and lactate levels.

### 2.3. Twenty-four-hour post-transfusion recovery (PTR24) of stored RBCs in healthy animals

Using technetium-99 radioactive label, each storage condition was tested for survival of viable RBCs 24 h. after transfusion as previously described [17]. 200 μL of the labeled blood was injected intravenously to Sprague-Dawley rats (*n* = 2 per storage condition). Blood samples were taken via tail clip at 5 min and 24 h. post-infusion. Blood samples were counted using a Cobra II gamma counter (Packard Instrument Co., Meriden, CT).

### 2.4. Animal preparation

Studies were performed in male Sprague Dawley rats weighing 350–400 g (Charles River Laboratories, Wilmington, MA). Animal handling and care followed the NIH Guide for Care and Use of Laboratory Animals, and all protocols were approved by the University of California San Diego Institutional Animal Care and Use Committee. All methods were carried out in accordance with the ARRIVE guidelines (Animal Research: Reporting of In Vivo Experiments). The control group was comprised of healthy animals who were not instrumented or injured (healthy control). Animal preparation, TBI, and HS methods were previously described [18]. Briefly, animals were anesthetized using isoflurane 5 % in compressed room air for induction (Drägerwerk AG, Lübeck, Germany) and then maintained at 1.5 %, except during surgical procedures when it was increased to 2.5 %. Animals were placed on a heating pad to preserve core body temperature at 37 °C and allowed to freely breathe from a nosecone delivering anesthesia. Animals were instrumented with a right femoral artery and vein catheter for hemodynamic assessment, and blood withdrawal and intravenous infusion, respectively. Animals were allowed to stabilize for 10 min and baseline measurements were collected.

### 2.5. Blunt traumatic brain injury

Animals were then moved to a stereotaxic apparatus and placed in the ventral position, and the isoflurane was increased to 2.5 % for 5 min before inducing TBI. To induce TBI, a 5 mm craniotomy was performed over the right cerebral cortex, and the dura was impacted with a 5.0 mm flat tipped impactor at a velocity of 5 m/s and dwell time of 200 ms via a pneumatically controlled cortical impactor (CCI; Leica Biosystems, Vista, CA). After CCI, the head was closed and the animals were placed back on the heating pad dorsally, and the isoflurane was decreased to 1.5 vol% for 10 min before starting hemorrhagic shock (HS).

### 2.6. Hemorrhagic shock

All animals were given 10 min to stabilize after performing the TBI before performing HS. Animals were intravenously heparinized (100 IU/kg) to ensure patency of the catheters during the protocol. Hemorrhage was induced by removing blood from the femoral vein (0.5 mL/min) until the Mean Arterial Pressure (MAP) reached 40 mmHg. The MAP was maintained between 35 and 40 mmHg for 90 min by withdrawing or returning small volumes of blood when MAP was out of the indicated range for >2 min. After the prolonged severe hypovolemia, animals were randomly assigned to one of three groups (*n* = 9/group) based on the test intravenous resuscitation fluid: Fresh red blood cells (FRBCs); Conventionally stored red blood cells (CRBCs) or Hypoxically stored blood cells (HRBCs). All RBCs suspensions were administered intravenously at 2 mL/min until it reached 70 % of the blood withdrawn during the HS period (typically around 7 mL). Animals were monitored for 120 min from the beginning of the resuscitation until euthanasia. Blood samples were taken at baseline (BL), 90 min into HS (HS), and 30 min and 2 h after resuscitation. A representative timeline for the experimental protocol is shown in Supplemental Fig. 1.

### 2.7. Systemic parameters

Arterial pressure and heart rate (HR) were recorded continuously from the femoral artery (MP150, Biopac, Santa Barbara, CA) at a 2 kHz sampling rate. Blood pressure recordings were used to calculate online MAP, systolic blood pressure (SBP), and diastolic blood pressure (DBP) using AcqKnowledge software (Biopac). Hematocrit was measured from centrifuged arterial blood samples taken in heparinized capillary tubes. Arterial and venous blood was collected in heparinized glass capillary tubes (65 μL) and immediately analyzed for oxygen partial pressure (PO_2_), carbon dioxide partial pressure (PCO_2_), pH, SO_2_, glucose, and lactate (ABL90; Radiometer America, Brea, CA).

### 2.8. Harvesting tissues

Two hours after reperfusion, 10mL of blood was collected from the indwelling arterial catheter and centrifuged to separate the plasma. Rats were euthanized with Euthasol (sodium pentobarbital, 300mg/kg), and urine, kidneys, liver, spleen, heart, and lungs were harvested. Markers of inflammation, organ function, organ injury, and oxidative stress were evaluated. These analyses were performed by the UC San Diego Histology Core with ELISA and flow cytometric analysis of tissue homogenates and plasma. The kits and methods used for these analyses are described in Supplementary Table 1.

The study measured neutrophil count from bronchoalveolar lavage fluid (BAL) analysis. Briefly, the left lung was lavaged with 5-mL isotonic sodium chloride solution at 37 °C. the BAL samples were centrifuged at 1500 rpm for 5 min at 10 °C. The pellet was resuspended in 200 μL of PBS; after that, 20 μL BAL fluid was mixed with Turks suspension. The neutrophil cell count was determined using a hemocytometer.

Myeloperoxidase (MPO) activity in homogenates of lung samples was measured. Lung tissues from each animal were homogenized, and the resulting homogenates were cleared and stored. Protein content was determined, and the samples were subjected to a reaction with the substrate o-dianisidine dihydrochloride. This reaction was carried out in a 96-well plate, and MPO activity was determined by measuring light absorbance at 460 nm. Standard MPO was used to create a curve for determining MPO activity in the samples. The process followed established protocols, and all reagents were purchased from Sigma Aldrich.

### 2.9. Statistical analysis

All values are expressed as mean ± SE. Data with multiple timepoints were analyzed using Two-Way Analysis of Variance (ANOVA) for repeated measurements. Tissues and plasma markers evaluated only after reperfusion were analyzed using one-Way Analysis of Variance (ANOVA). When appropriate, post hoc analyses were performed with the Tukey multiple comparisons test. All statistics were calculated using Graph-Pad Prism 6 (GraphPad Software, Inc., San Diego, CA). Results were considered significant if *p* < 0.05.

## 3. Results

### 3.1. Properties of stored RBCs at transfusion: oxygen reduction and PTR24

**Fig. 1.**
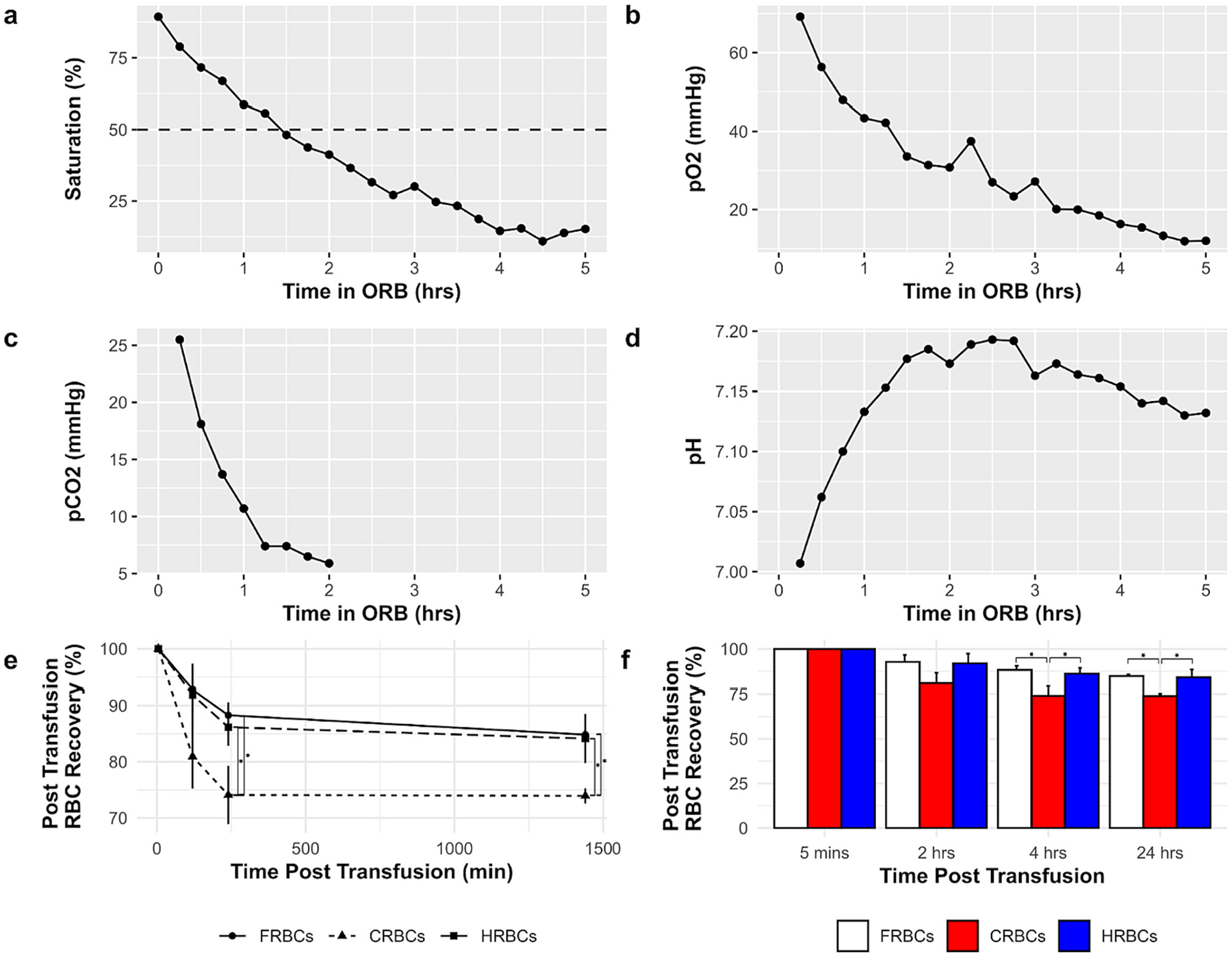
Oxygen Reduction Bag (ORB) changing blood gases (*n* = 1): a) oxygen saturation (sO2), b) oxygen tension (pO2), c) carbon dioxide tension (pCO2), d) pH. Panels e) and f) show RBCs post transfusion into rats over a 24-hour period (*n* = 3 per group). * *p*< 0.05 between groups.

Changes in blood gases for the HRBCs are shown in Fig. 1, panels a–d. Over the 5 h of processing, the SO_2_ decreased from 89.4 % to 15.2 %, while pO_2_ decreased from 69.2 mmHg to 12 mmHg. The pCO_2_ decreased from 25.5 mmHg to <5 mmHg within 2 h, at which point the levels of CO_2_ were outside the range detectable by the ABL90 blood gas analyzer machine. The pH increased from 7.007 to 7.1 over 5 h due to CO_2_ reduction.

The percentages of viable RBCs 24 h after transfusion into healthy animals are shown in Fig. 1, panels e and f. At both 4 h. and 24 h. post-transfusion, the *PTR24 for* CRBCs (73.9 % ± 1.4 %) was significantly lower than both the FRBCs (84.8 % ± 0.9 %) and the HRBCs (84.2 % ± 4.4 %). (Fig. 1). The properties of blood used for transfusion for all groups are shown in Supplemental Fig. 2. The pCO_2_, pO_2_, and SO_2_ were all significantly lower in the HRBCs group as compared to both the FRBCs and CRBCs. The pH in the FRBCs was significantly higher than both the CRBCs and the HRBCs. There were no significant differences in total hemoglobin (tHb), hematocrit (Hct), or % hemolysis.

### 3.2. In vivo hemodynamics

**Fig. 2.**
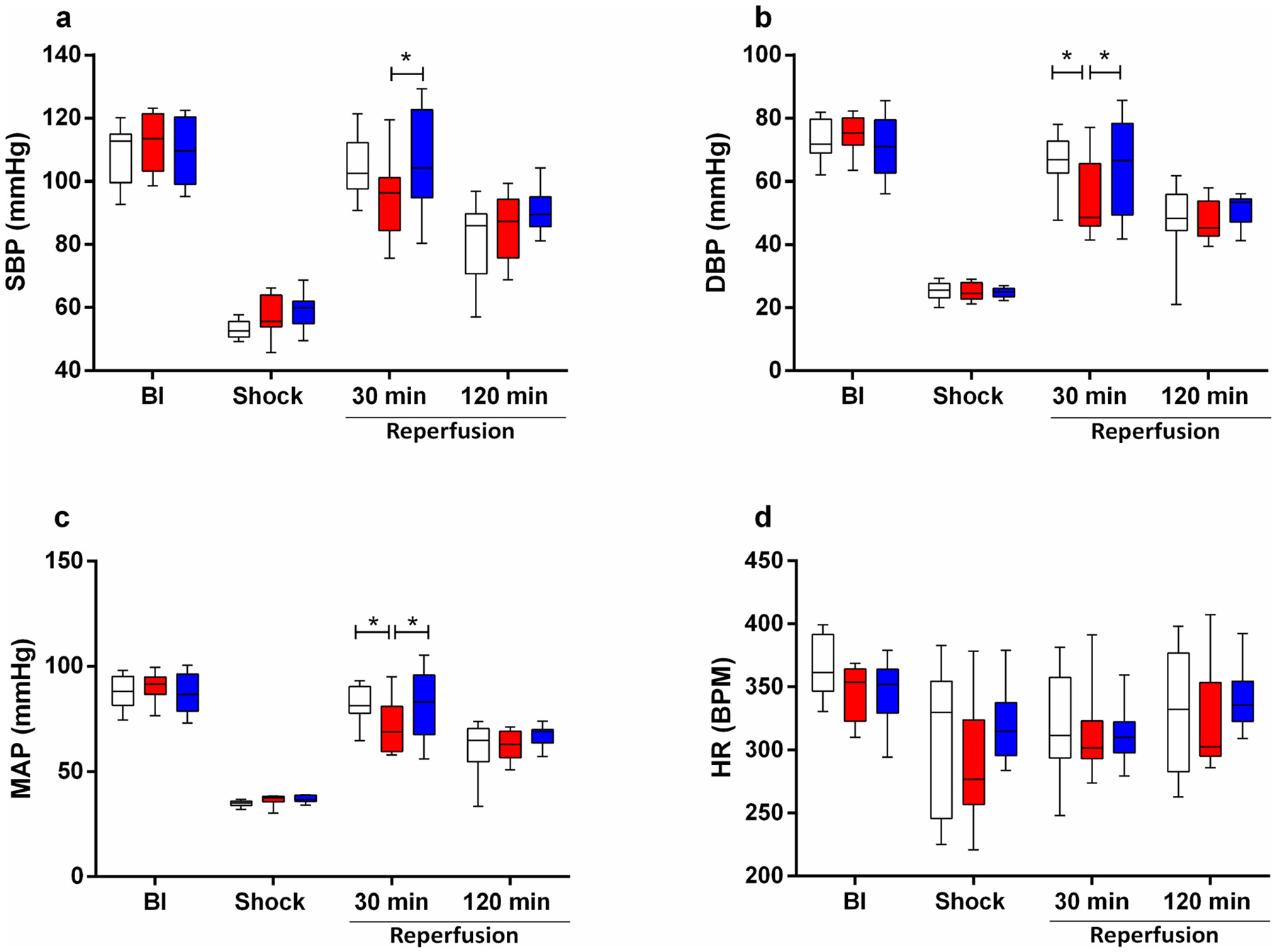
Blood Pressure measurements of Fresh red blood cells (FRBCs); Conventionally stored red blood cells (CRBCs); and Hypoxically stored red blood cells (HRBCs). a- Systolic blood pressure (SBP); b- Diastolic blood pressure (DBP); c- Mean arterial pressure (MAP); d- Heart Rate (HR). At Baseline (BL), Hemorrhagic Shock (Shock) and 30 and 120 min after reperfusion. *p < 0.05. FRBCs (*n* = 9); CRBCs (*n* = 8); HRBCs (n = 9).

To evaluate the use of HRBCs to restore hemodynamics, we used a severe model of TBI followed by HS, resuscitated the animals with HRBCs, and compared the BP restoration after resuscitation with FRBCs or CRBCs. Most changes in hemodynamics were observed 30 min into resuscitation as shown in Fig. 2. At this timepoint, resuscitation with HRBCs restored blood pressure similarly to FRBCs with MAP, SBP and DBP being restored relative to baseline 93 %, 96 % and 90 % respectively, while CRBCs restored MAP, SBP and DBP to 84 %,72 % and 79 % respectively. No differences in heart rate (HR) were observed between groups. (Fig. 2d). At 120 min all hemodynamics parameters were similar, and MAP approximated 70 % for FRBCs, 77 % for HRBCs and 68 % for CRBCs relative to baseline.

Animals receiving HRBCs and FRBCs had higher pO_2_ ([Supplementary Table 2) compared to CRBCs. There were no differences in pH, pCO_2_, O_2_ saturation, hematocrit, and total hemoglobin between groups 30 min into resuscitation, and there were no changes in any measurements 2 h after resuscitation. Finally, lactate increased equally between groups during HS, and decreased similarly after reperfusion remaining slightly higher than baseline in all groups (Supplementary Table 2).

### 3.3. Organ damage and function

#### 3.3.1. Lung

**Fig. 3.**
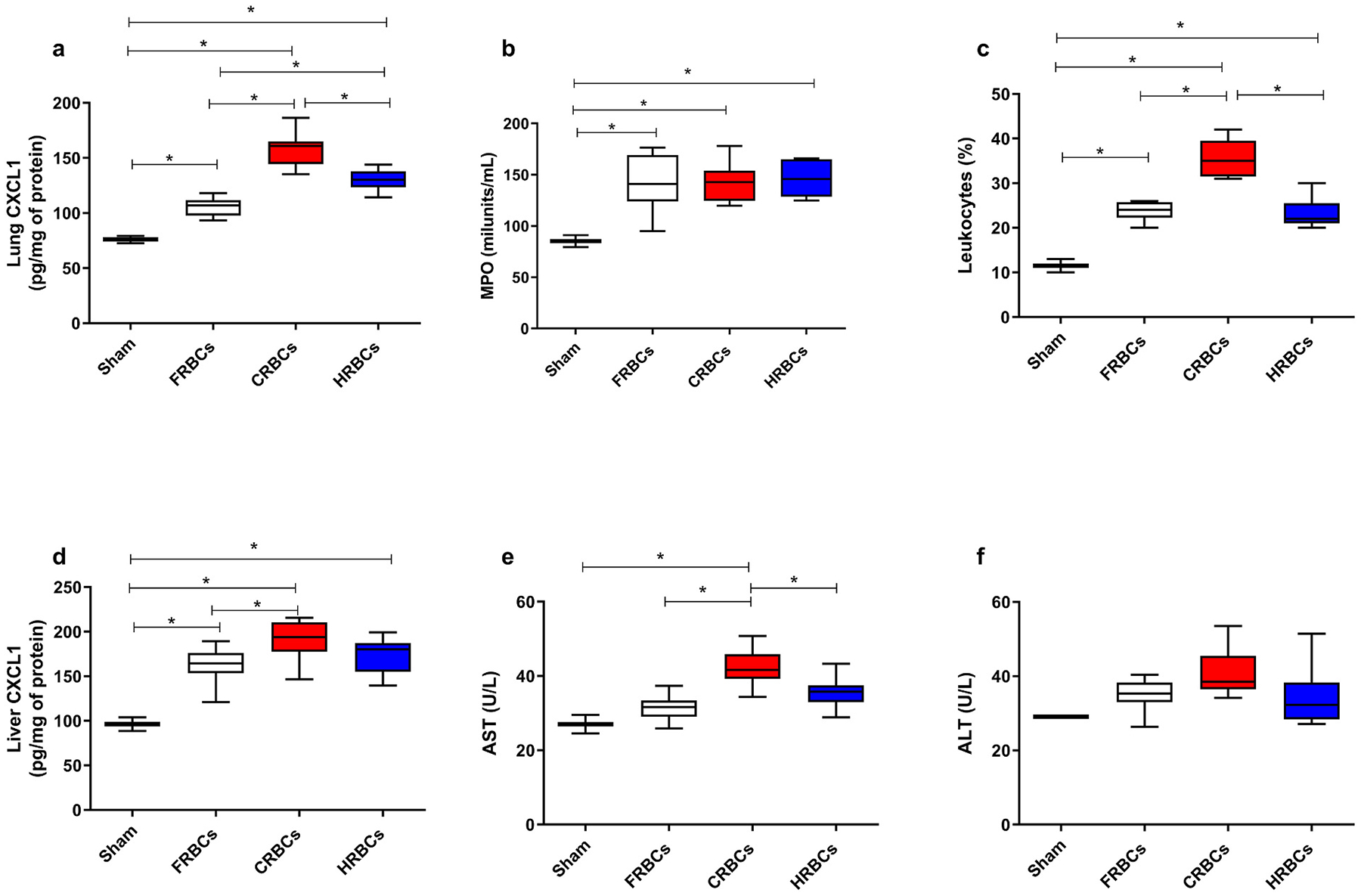
Liver and lung injury after reperfusion with of Fresh red blood cells (FRBCs); Conventionally stored red blood cells (CRBCs); and Hypoxically stored red blood cells (HRBCs). a-Lung chemokine ligand- 1 (CXCL-1); b -Myeloperoxidase (MPO) and c- Leukocytes levels in the lungs, markers of lung injury. d- Liver CXCL-1; e-Aspartate aminotransferase (AST); f- Alanine aminotransferase (ALT), 120 min after reperfusion. *p < 0.05. FRBCs (n = 8); CRBCs (n = 9); HRBCs (n = 9).

To assess lung inflammation and injury we evaluated chemokine ligand 1 (CXCL-1) (Fig. 3a), myeloperoxidase (MPO) (Fig. 3b) and Leukocytes count (Fig. 3c) in the lungs. It was observed that all the groups post-resuscitation presented higher levels of CXCL1 and MPO and Leukocytes count compared to healthy control, however CXCL1 and Leukocytes count level was even higher for the CRBCs compared to FRBCs and HRBCs. CXCL1 level was slightly higher in the HRBCs group compared to FRBCs.

#### 3.3.2. Liver

Following resuscitation, the FRBCs group had the smallest increase in liver CXCL1 while all groups demonstrated elevations significantly higher than healthy control (Fig. 3d) Aspartate aminotransferase (AST) levels were lower in FRBCs and HRBCs compared to CRBCs (Fig. 3e), and there were no significant elevations of Alanine aminotransferase (ALT) in any group compared to control. (Fig. 3f).

#### 3.3.3. Cardiac

**Fig. 4.**
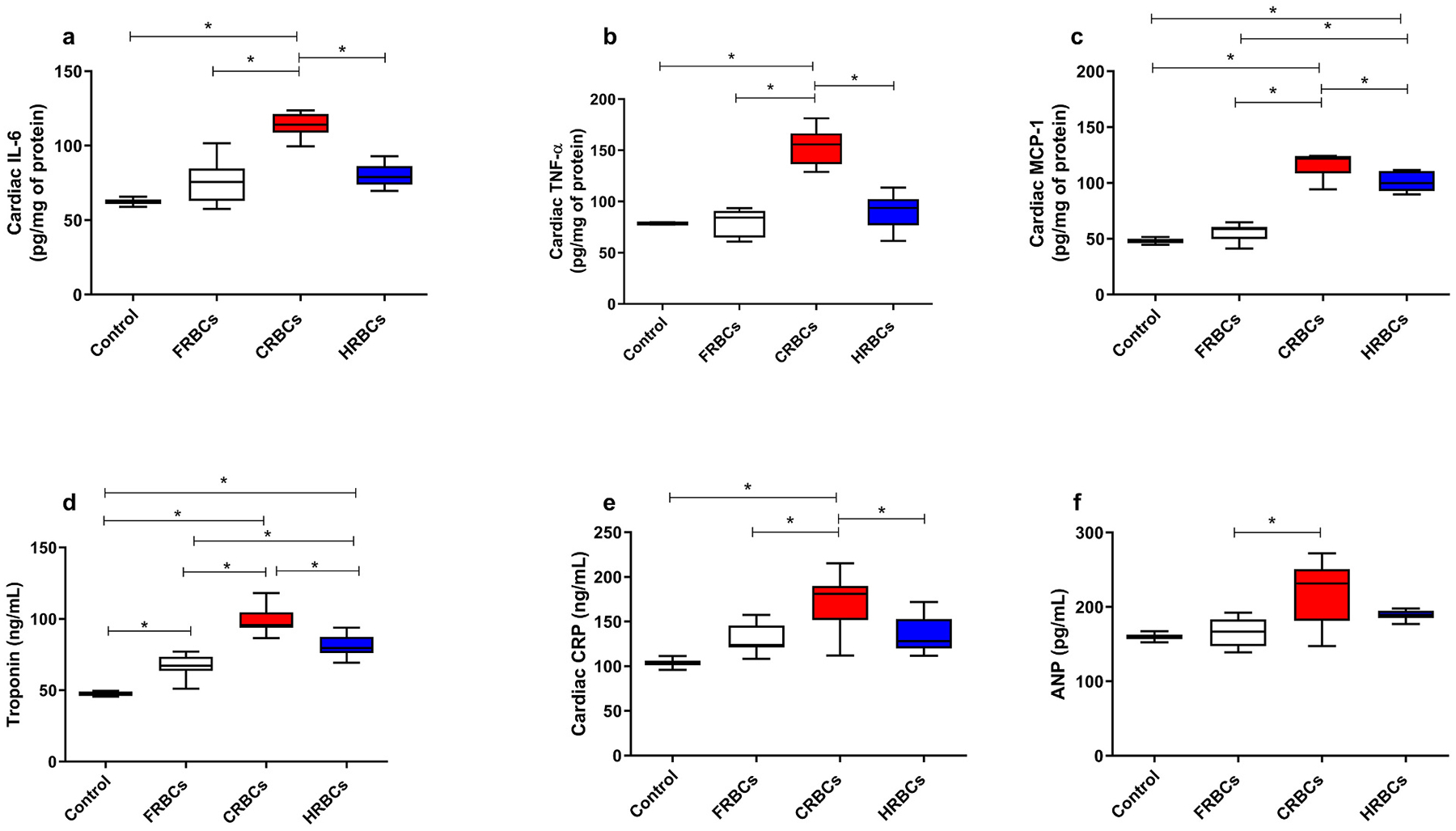
Cardiac inflammation and injury after reperfusion of Fresh red blood cells (FRBCs); Conventionally stored red blood cells (CRBCs); and Hypoxically stored red blood cells (HRBCs). a- Cardiac interleukin- 6 (IL-6); b- Cardiac tumor necrosis factor alpha (TNF-α); c- Cardiac monocyte chemoattractant protein-1 (MCP-1). d- Cardiac troponin; e- C-reactive protein; f- Atrial natriuretic peptide (ANP), 120 min after reperfusion. *p < 0.05. FRBCs (n = 8); CRBCs (n = 9); HRBCs (n = 9).

A set of markers was investigated to quantify cardiovascular damage. The group transfused with CRBCs displayed higher levels of Interluekin-6 (IL-6) (Fig. 4a), Tumor Necrosis Factor alpha (TNF-alpha) (Fig. 4b), and cardiac C-Reactive protein (CRP) (Fig. 4e) as compared to all the other groups. Monocyte Chemoattractant Protein-1 (MCP-1) (Fig. 4c) and cardiac troponin (Fig. 4d) were increased for both CRBCs and HRBCs when compared to FRBCs and control groups. Importantly, HRBCs presented lower levels for both markers compared to CRBCs. ANP (Fig. 4f) levels were higher for CRBCs compared to FRBCs.

#### 3.3.4. Kidney

To assess kidney damage, serum and urine creatinine and urinary neutrophil-associated lipocalin (U-ngal) were measured, and no differences were observed among groups. Blood urea nitrogen (BUN) was higher for both CRBCs and HRBCs compared to FRBCs. No post-mortem changes were observed when compared to healthy control, suggesting no acute kidney injury was associated with this HS and TBI model (Table 1).

#### 3.3.5. Systemic inflammatory activation

**Fig. 5.**
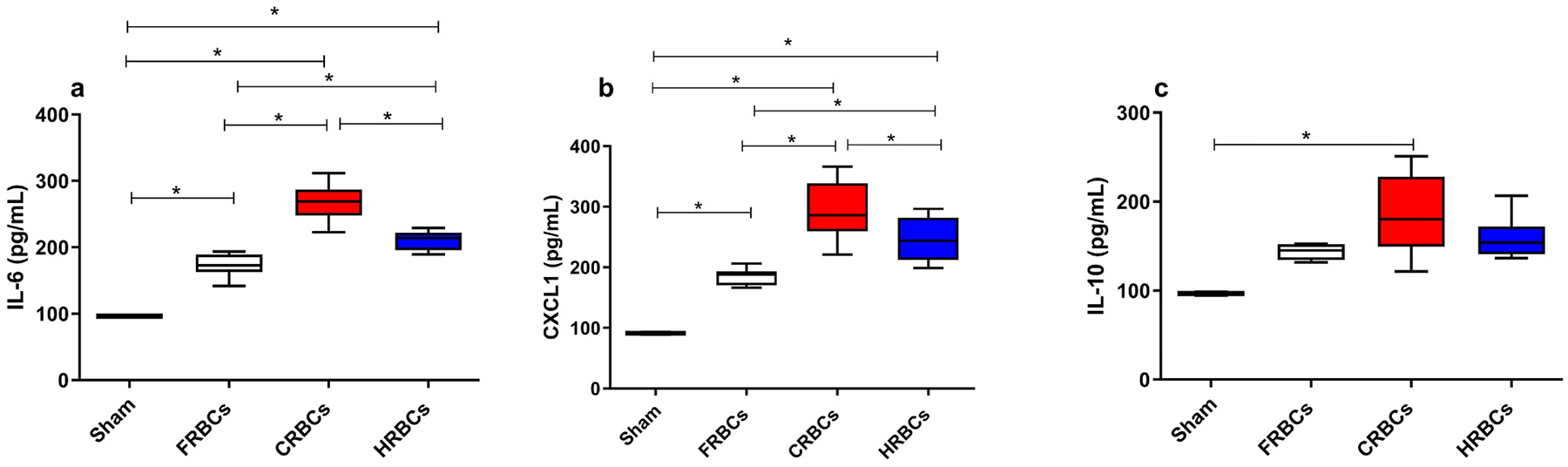
Systemic inflammation after reperfusion of Fresh red blood cells (FRBCs); Conventionally stored red blood cells (CRBCs); and Hypoxically stored red blood cells (HRBCs). a- Plasmatic interleukin- 6 (IL-6); b- Plasmatic chemokine ligand- 1 (CXCL-1); c- Plasmatic interleukin- 10. 120 min after reperfusion. *p < 0.05. FRBCs (n = 8); CRBCs (n = 9); HRBCs (n = 9).

Inflammation is classically expected after transfusion, especially with stored blood. In this study we evaluated serum IL-6, CXCL1 and Interleukin-10 (IL-10) and observed that IL-6 and CXCL1 were higher for the 3 post-transfusion groups when compared to healthy control, but CRBCs presented higher levels of both markers when compared to FRBCs. Although HRBCs still had higher levels of IL-6 and CXCL1 compared to FRBCs, these levels were lower compared to CRBCs. IL-10 was higher only in the CRBCs compared to healthy control. (Fig. 5).

#### 3.3.6. Systemic oxidative stress

**Fig. 6.**
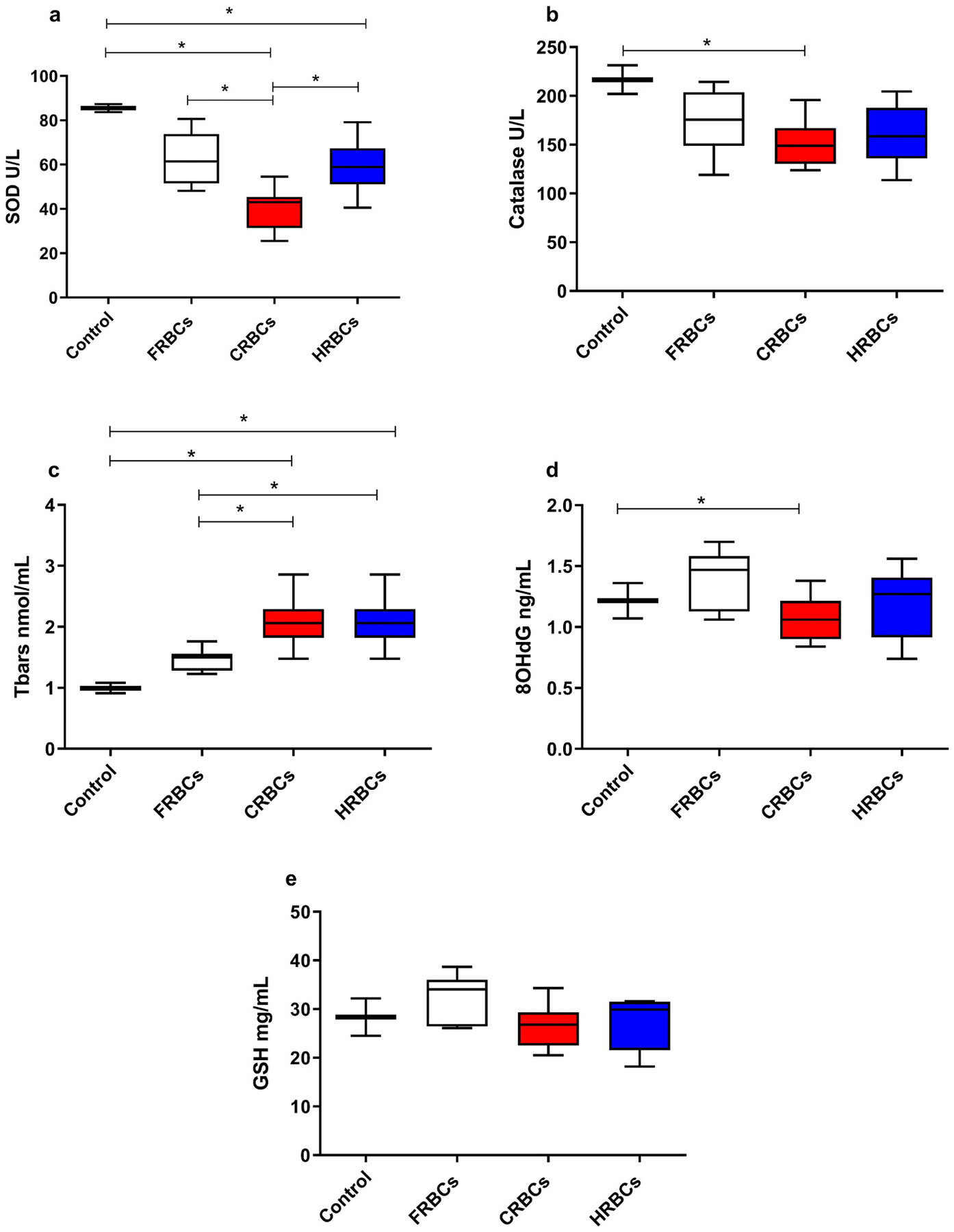
Oxidative stress after reperfusion with of Fresh red blood cells (FRBCs); Conventionally stored red blood cells (CRBCs); and Hypoxically stored red blood cells (HRBCs). a-Superoxide dismutases (SOD); b- Catalase; c-Thiobarbituric Acid Reactive Substances (TBARS). d- 8-hydroxy-2′-deoxyguanosine (8-OHdG); e- Glutathione (GSH), 120 min after reperfusion. *p < 0.05. FRBCs (n = 8); CRBCs (n = 9); HRBCs (n = 9).

We studied representative biomarkers for oxidative stress (catalase, Superoxide dismutase [SOD] and Glutathione [GSH]), lipid peroxidation (thiobarbituric acid reactive substances [Tbars]), and ribonucleic acid [RNA] and deoxyribonucleic acid [DNA] injury 8-hydroxylation of guanine [8-OhdG] after reperfusion with CRBCs, FRBCs and HRBCs (Fig. 6).

We observed that SOD (Fig. 6a) decreased post-transfusion compared to healthy control, independent of the solution used; however, the levels of SOD were even lower in CRBCs compared to FRBCs and HRBCs. Catalase (Fig. 6b) and 8OHdG (Fig. 6d) only decreased in the CRBCs compared to control. Moreover, Tbars increased in the animals reperfused with CRBCs and HRBCs compared to both healthy control and FRBCs (Fig. 6c). Finally, no changes in GSH were observed among groups (Fig. 6e).

## 4. Discussion

In the last few years, new methods have been proposed to improve the quality of RBCs during storage based on the hypothesis that oxygen-mediated oxidative stress drives development of RBCs storage lesions [19]. D’Alessandro et al. demonstrated that hypoxically stored RBCs possess superior post-transfusion recovery, comparable hemolysis to donor-paired standard units, and better energy and redox metabolism [20]. Hypoxically stored RBCs have been shown to increase 2,3 DPG and ATP levels up to 21 and 42 days of storage compared to conventional RBCs, and are characterized by a higher P50, indicating improved oxygen release to the tissues [21,22]. Furthermore, it has been reported that hypoxic blood storage is capable of considerably decreasing the amount of non-deformable cells present in the overall population of relatively well-preserved RBCs [23,24].

Prior work from this lab demonstrated that rats subjected to HS who were resuscitated with anaerobic RBCs were able to reach 90 % baseline MAP (primary endpoint) in less time and with half as much blood volume as rats who received conventional blood. Lactate was cleared faster with hypoxically stored blood and was close to normal range after 60 min of resuscitation. Additionally, markers of kidney damage (BUN, u-NGAL) were reduced, and necropsy revealed less ischemic damage to the organs of rats who received anaerobic vs conventional RBCs [17]. The addition of TBI to HS creates a more clinically challenging condition. A secondary analysis from the Pragmatic, Randomized Optimal Platelets and Plasma Ratios (PROPPR) trial found [25] that the combination of TBI + HS occurred in 18 % of the patients in this multi-center study. The PROPPR study compared patients with TBI + HS to TBI alone and HS alone and found that admission vital signs and perfusion indices were significantly different in TBI + HS. Specifically, TBI + HS patients had lower systolic blood pressure and higher heart rates compared to other groups as well as higher lactate levels than TBI alone. Additionally, patients with TBI + HS were transfused with more RBCs, FFP, and platelets compared to all other groups. This was associated with an overall worse coagulopathy. Finally, TBI + HS patients were found to have increased 30-day mortality and increased chance of lung complications.

While fluid resuscitation restores perfusion to ischemic tissues and prevents hypoxic and ischemic damage induced by HS, it also has the potential to worsen TBI-related brain pathology leading to cerebral edema, diffuse swelling, and elevated intracranial pressure (ICP). Loss of local and systemic autoregulatory mechanisms can limit the recovery of hemodynamics after resuscitation [26,27]. Cerebral edema leading to high intracranial pressure and decreased cerebral perfusion pressure is one of the most prominent pathophysiological factors associated with death and unfavorable outcomes after TBI. Therefore, treatment of TBI + HS is based on balanced transfusion of RBCs, plasma and platelets which has been shown to support microcirculatory perfusion, improve tissue oxygenation, and preserve mitochondrial function and cerebral blood flow after TBI [28–30]. In light of this evidence, it is highly important to treat TBI followed by HS with high quality RBCs to potentially reduce secondary brain injury and improve patient outcomes.

For this second experiment, twenty-four-hour post transfusion recovery (PTR24) of stored RBCs in healthy animals was first assessed because it is the Food and Drug Administration (FDA)-approved metric for determining the acceptability of stored RBCs in additive solutions. Recovery of viable cells must be >75 % to comply with FDA blood product requirements [15]. Studies have identified that, after storage, a variable proportion of transfused RBCs are removed from the circulation in the first 24h following transfusion. The proportion of transfused RBCs that remain in circulation is an important surrogate marker of transfusion efficacy [31]. In the prior HS study from this group [17], rat RBCs stored in hypoxic/hypocapnic conditions demonstrated increased PTR24 and reduced in-storage hemolysis compared to conventional storage. In the current study, we again observed that, after 3 weeks of storage, HRBCs and FRBCs had significantly higher PTR24 (84 % and 85 % respectively) compared to CRBCs (74 %), suggesting that conventionally stored RBCs accumulate storage-induced damages following blood collection and processing and confirming that hypoxic storage improves function and quality of rat RBCs.

The experimental methodology compared outcomes in rats subjected to TBI + HS who were administered either RBCs that were conventionally processed and stored at 4 °C for 3 weeks, RBCs that were processed to reach an SO_2_ < 10 % and then stored hypoxically at 4 °C for 3 weeks, and fresh RBCs that were transfused within 2 days from collection. The study was designed as a pilot with short-term (2 h) outcomes to fine-tune an appropriate model for TBI + HS so that a more detailed examination of the effect of hypoxic blood on the injured brain can be performed. We decided to target volume during resuscitation and infused 70 % of the shed blood withdrawn during HS. This decision was made based on a previous study where 70 % of shed fresh blood was enough to resuscitate the animals and resulted in an approximate MAP of 75 mmHg at 2 h after reperfusion [18]. Targeting volume in a model of TBI + HS has value since infusing higher volumes in order to achieve an ideal MAP could exacerbate edema due to loss of autonomic control and increased blood pressure variability that occurs with TBI. These alterations make it difficult to control and maintain safe blood pressure levels during resuscitation from HS after TBI. Additionally, it has been shown that large resuscitation volumes increase the risk of cerebral edema and worsen TBI-associated clinical sequelae [32] whereas a balanced blood product resuscitation has been shown to be beneficial [33,34].

In the present study, resuscitation from TBI + HS with 3-week-old HRBCs improved hemodynamics as well as FRBCs. At the 30-min timepoint in resuscitation, blood pressure recovery (MAP) was comparable between the FRBCs and HRBCs groups and significantly higher in the FRBCs and HRBCs groups compared to the CRBCs group. Hypoxic blood also was seen to rapidly reduce lactate levels. Finally, pO_2_ was significantly lower in the CRBCs group at 30 min compared to FRBCs and HRBCs, indicating a correlation between the oxygen tension post-transfusion and the lack of blood pressure restoration. Furthermore, these results indicate that reduced levels of O_2_ during storage of HRBCs did not have a negative impact on the restoration of pO_2_ levels and supports that pre-oxygenation of HRBCs before transfusion is not necessary.

### 4.1. Organ system effects

Overall, resuscitation from TBI + HS with HRBCs resulted in significantly improved organ function and reduced vital organ injury compared to FRBCs and was superior to CRBCs. Transfusion with conventional blood can result in organ damage and inflammation as evidenced by elevated inflammatory markers in the lung and liver. In this study, CXCL1 and Leukocytes levels were higher for the CRBCs compared to FRBCs and HRBCs. These results suggest that storing RBCs hypoxically diminishes lung injury post-transfusion. In the liver, bilirubin and AST were lower in FRBCs and HRBCs compared to CRBCs. Furthermore, a set of cardiac markers (Interleukin-6 [IL-6]; Tumor Necrosis Factor alpha [TNF-α]; Monocyte Chemoattractant Protein-1 [MCP-1]; troponin; and C-Reactive Protein [CRP]) that indirectly demonstrate cardiac function and damage [35,36] showed substantial increases two hours post-transfusion with CRBCs, but not with HRBCs and FRBCs (Fig. 4). In particular, troponin and the pro-inflammatory cardiac biomarkers TNF-α and IL-6 were significantly lower in FRBCs and HRBCs compared to CRBCs and levels of TNF-α and IL-6 were actually comparable to control (non-injured) values. The CRBCs group exhibited a systematically higher-level IL-10, whereas the other groups did not demonstrate a statistically significant increase in this cytokine when compared to the health control. IL-10, being an anti-inflammatory protein, plays a pivotal role in preventing additional injury to target organs. Despite all groups displaying elevated inflammatory markers, the CRBCs also activates the anti-inflammatory pathway. Transfusion has been shown to affect immune function and can induce inflammatory responses. Previous studies have identified, but not quantified, a small number of chemokines associated with RBCs, supporting the hypothesis that, alongside its O_2_ delivery, RBCs play a role in cytokine signaling [37]. This discovery may help supplement disease biomarker research and may shed light on adverse inflammatory processes that can follow RBCs transfusion, further evaluations are imperative to compare the effect of transfusion of stored blood on inflammatory pathways.

### 4.2. Secondary brain injury

Resuscitation from TBI + HS in trauma patients is conducted to minimize primary and secondary brain injury which both result in morbidity and mortality [38,39]. The primary injury is caused by a mechanical force, which results in direct damage to the neurons, glial cells, and blood vessels. Secondary brain injury is the term given to the biochemical, cellular, and physiologic changes that evolve over a period of hours to days after the primary brain injury and these changes contribute to further destruction of brain tissue. Excitotoxicity, mitochondrial dysfunction, oxidative stress, lipid peroxidation, neuro-inflammation, axon degeneration and apoptotic cell death as well as DNA and RNA oxidative damage have all been described as secondary injuries in the traumatized brain [30,40].

We studied representative biomarkers for oxidative stress (catalase, SOD and GSH), lipid peroxidation (Tbars), and ribonucleic acid (RNA) and deoxyribonucleic acid (DNA) injury (8-OhdG) after reperfusion with CRBCs, FRBCs and HRBCs (Fig. 6). Oxidative stress cascades play a major role in development of secondary injury [41,42]. Reactive oxygen species (ROS), including superoxide (O2•−), hydrogen peroxide (H2O2), and hydroxyl radicals (•OH), produced by mitochondria and from cellular oxidative metabolism are several of the main pathological agents involved in the oxidative stress that characterizes TBI. Enzymes that remove superoxide and H_2_O2 protect the cell against oxidative stress. Catalase, glutathione peroxidase (GPx) and SOD are the major defense enzymes against superoxide radicals [43].

In this study we measured plasma SOD, catalase, and glutathione (GSH) as biomarkers of oxidative stress at 2 h after resuscitation. We found that, after reperfusion, the SOD levels in the HRBCs group were similar to the FRBCs group and both were significantly higher than CRBCs. Catalase levels were not different than control values in the FRBCs and HRBCs groups but significantly lower with CRBCs. Finally, no differences in GSH levels were seen between groups.

Thiobarbituric acid reactive substances (Tbars) were examined as biomarkers for lipid peroxidation and levels were higher for both CRBCs and HRBCs. The clinical data regarding Tbars in TBI outcome is conflicting – when Lorente and colleagues looked at a specific end-product of lipid peroxidation - serum malondialdehyde (MDA) - they found that non-survivors of TBI had higher (MDA) concentrations at days 1, 4 and 8 after TBI in their study of 118 patients. The non-surviving patients had significantly lower GCS than survivors (4 vs 8) [44].

On the other hand, Hohl et al. assayed 79 consecutive patients with severe TBI and found that plasma levels of Tbars increase significantly in the first 70 h after severe TBI but are not independently associated with hospital mortality by multiple logistic regression analysis [45].

Oxidative damage to ribonucleic acid (RNA) and deoxyribonucleic acid (DNA) has also been found to occur in TBI. Guanine is the most susceptible base to oxidative/nitrosative modifications because of its lowest reduction potential. The formation of 8-hy-droxy-2′-deoxyguanosine (8-OHdG) and 8-hydroxy-2′-guanosine (8-OHG) are the most studied interactions of hydroxyl radicals with guanine in DNA and RNA, respectively [46]. Lorente et al. found significantly higher serum concentrations of OGS (8-OHdG from DNA, 8-hydroxyguanosine from RNA, and 8-hydroxyguanine from DNA or RNA) in non-surviving (*n* = 34) than in surviving patients (*n* = 90), and an association between serum OGS levels and 30-day mortality after control for CGS, age, and computed tomography findings [40]. In this study, there were no differences in 8-OHdG levels compared to control values when resuscitated with FRBCs or HRBCs, although the lowest levels occurred with CRBCs. It is important to consider that the decrease in the 8-OHdG is a positive result meaning that there is no oxidation of nucleic acids after injury, and further evaluations are necessary to better understand the full pathology on our model.

## 5. Limitations

The present study was conducted in anesthetized animals, and isoflurane has potent dose-dependent cardiovascular and cerebral effects, causing myocardial depression, vasodilation, and HR depression. Isoflurane also induces cerebral vasodilation, increases cerebral blood flow, decreases cerebral metabolism, and impairs cerebral blood flow autoregulation [47]. However, the implication of isoflurane to the results of the current study was equal for all experimental groups as all animals were subjected to an equal dose of isoflurane during the protocol.

Furthermore, it is worth noting that no assessment of oxidative stress markers in the blood was conducted before transfusion in the animals. This aspect will be investigated in the subsequent study as it holds significance in determining the oxidative stress status of RBCs and consequently evaluating the extent of storage-related aging damage. Additionally, a follow-up study will be conducted to evaluate the degree of brain damage after reperfusion, utilizing functional testing, now that the model has demonstrated survivability.

In summary, this study employed a novel oxygen reduction bag (ORB) to remove O_2_ and CO_2_ from collected blood, resulting in rapid reduction of pO_2_, pCO_2_, and SO_2_ prior to storage. By storing hypoxically stored RBCs (HRBCs) in an oxygen barrier bag, the initial hypoxic condition was maintained throughout the entire storage period. Resuscitation using anaerobically stored RBCs in cases of TBI + hemorrhagic shock swiftly restored hemodynamics, reduced organ injury, and partially alleviated oxidative stress post-resuscitation by decreasing SOD. Furthermore, anaerobically stored blood obviated the need for reoxygenation before infusion, effectively restoring oxygenation. Further research will delve into the impact of hypoxic blood on cognitive performance in animals following TBI + HS.

## Supplementary Material

supplemental

## Figures and Tables

**Table 1:** Kidney injury, and catecholamines.

	Control	FRBCs	CRBCs	HRBCs
U-ngal (ng/ml)	N/A	595 ± 38.14	703.3 ± 32.88	718.5 ± 56.01
Serum creatinine (mg/dL)	1.30 ± 0.20	1.73 ± 0.11	1.77 ± 0.10	1.68 ± 0.11
Urine creatinine (mg/dL)	N/A	1.38 ± 0.12	1.56 ± 0.08	1.42 ± 0.11
BUN (mg/dL)	1.08 ± 0.06	0.84 ± 0.05	1.17 ± 0.02*	1.07 ± 0.05*
Norepinephrine (pg/mL)	268.1 ± 36.3	349.5 ± 9.5	381.8 ± 19.4^‡^	330.6 ± 8.5
Epinephrine (pg/mL)	267.6 ± 39.3	402.6 ± 28.2	520.0 ± 26.2^‡^*	405.7 ± 14.2^‡^

Data presented as mean ± SE.

‡ p < 0.05 compared to control;

* p < 0.05 compared to FRBCs

† < 0.05 compared to CRBCs. control (*n* = 2); FRBCs (n = 8); CRBCs (n = 9). Fresh red blood cells (FRBCs); Conventionally stored red blood cells (CRBCs); Hypoxically stored red blood cells (HRBCs).

## Appendix A. Supplementary data

Supplementary data to this article can be found online at https://doi.org/10.1016/j.lfs.2024.122423.
