# Supplementary material for: Hypoxically stored RBC resuscitation in a rat model of traumatic brain injury and severe hemorrhagic shock
Source: Life Sci. Author manuscript; Available in PMC 2026 Jan 14. (PMC12802500; doi:10.1016/j.lfs.2024.122423)
Supplement: supplemental [file NIHMS2136046-supplement-supplemental.docx]

**LFS 122423**

**Hypoxically Stored RBC Resuscitation in a Rat Model of Traumatic Brain Injury and Severe Hemorrhagic Shock**

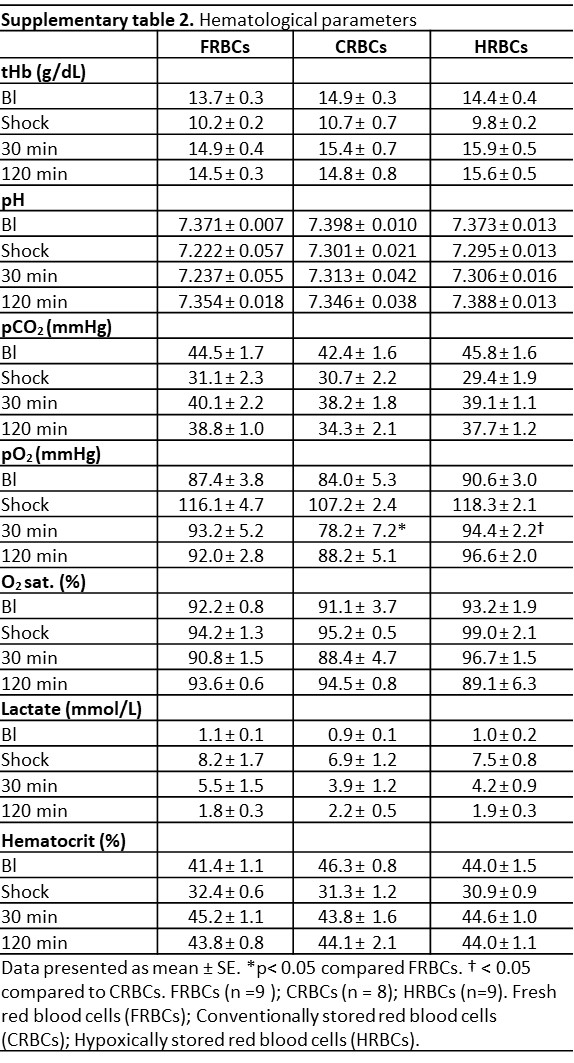


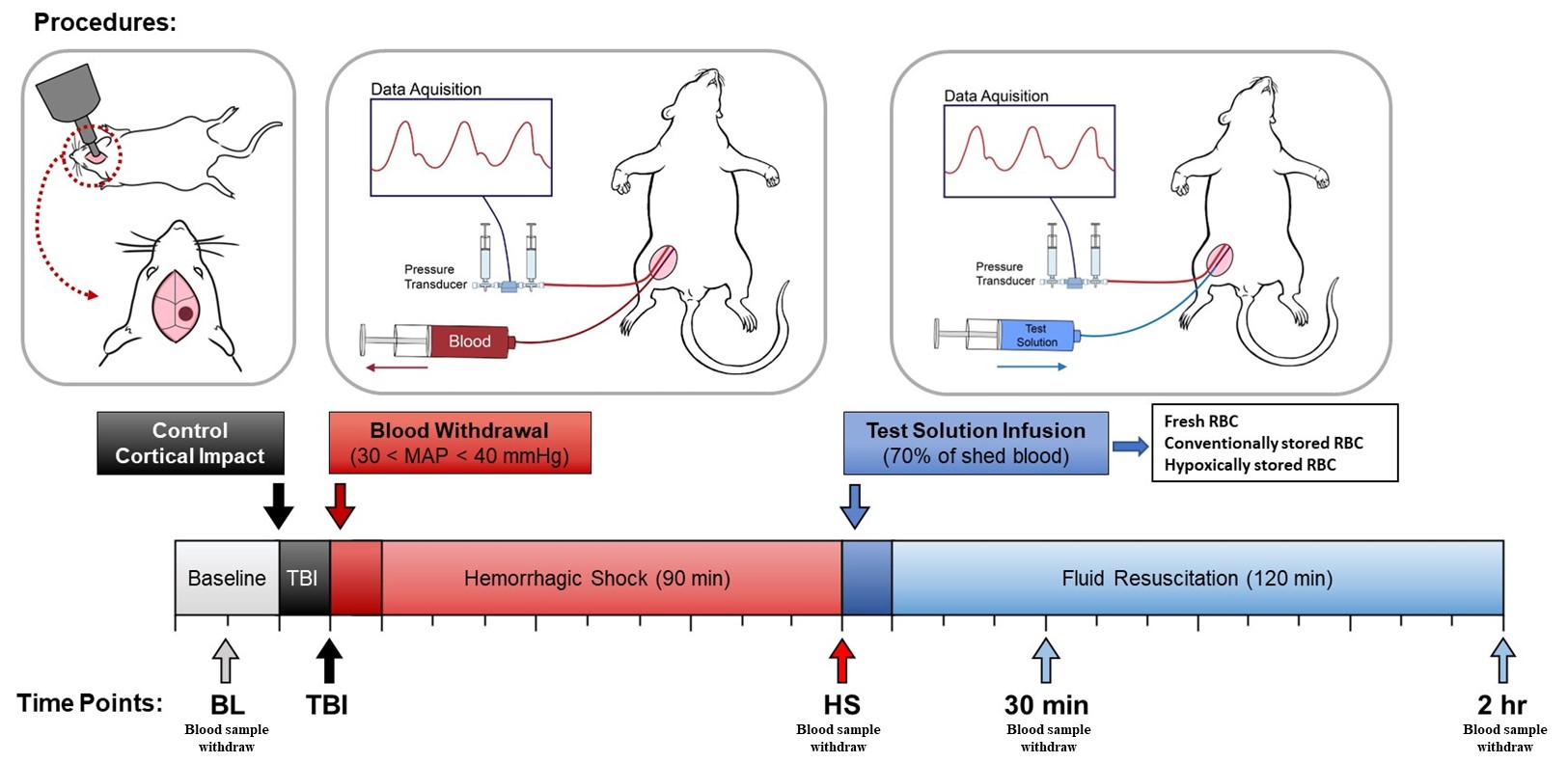


**Supplementary Figure 1**: Representative timeline for the experimental protocol.


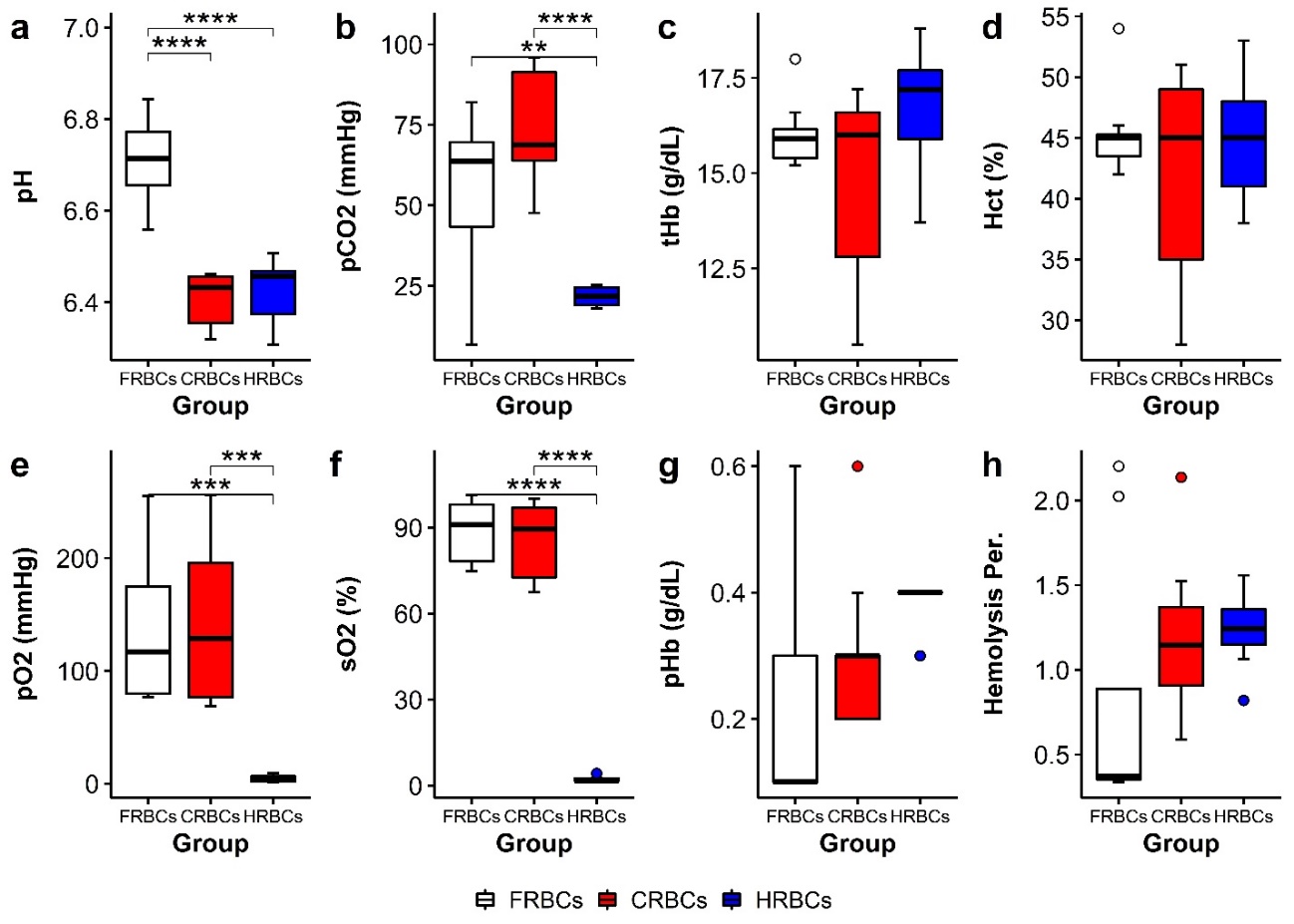


**Supplementary Figure 2.** Differences in blood used for resuscitation. a) pH, b) pCO2, c) total Hb, d) Hct, e) pO2, f) sO2, g) pHb, h) hemolysis percentage. FRBCs (n =8 ); CRBCs (n = 9); HRBCs (n=9). *** = P<0.05. **  = P<0.01. *** = P<0.001. **** = P<0.0001 between each storage group.**

**Supplementary Figure 3.** Mean arterial pressure (MAP) 10-minutes interval of Fresh red blood cells (FRBCs); Conventionally stored red blood cells (CRBCs); and Hypoxically stored red blood cells (HRBCs).. ***p< 0.05 CRBCs vs. FRBCs and HRBCs**. FRBCs (n =8 ); CRBCs (n = 9); HRBCs (n=9).
